# Nursing strategies in antimicrobial stewardship in the hospital environment: a qualitative systematic review

**DOI:** 10.1186/s12912-024-01753-y

**Published:** 2024-03-01

**Authors:** Flavia Giron Camerini, Tonia Lourenço Cunha, Cintia Silva Fassarella, Danielle de Mendonça Henrique, Juliana Gerhardt Soares Fortunato

**Affiliations:** 1 Medical-Surgical Department of the Faculty of Nursing, State University of Rio de Janeiro, Boulevard 28 de Setembro, 157 - Vila Isabel, Rio de Janeiro, RJ 20551-030 Brazil; 2 Faculty of Nursing, State University of Rio de Janeiro, Rio de Janeiro, RJ Brazil

**Keywords:** Anti-bacterial agents, Drug resistance, Nursing care, Review

## Abstract

**Background:**

Antimicrobial resistance has become one of the world’s most important public health problems. Accordingly, nursing strategies to manage antimicrobials in hospital environments are fundamental to promoting patient health. The aim of this study was to summarise the best evidence available on nursing strategies for the safe management of antimicrobials in hospital environments.

**Methods:**

This qualitative systematic review used meta-aggregation in accordance with the recommendations of the Joanna Briggs Institute. The protocol was registered in the data base of the Prospective Register of Systematic Reviews under No. CRD42021224804. The literature search was conducted, in April and May 2021, in the following data bases and journal repositories: Latin American and Caribbean Health Sciences Literature (LILACS) via the Virtual Health Library (VHL), Medical Literature Analysis and Retrieval System on-line (Medline) via PubMed, Cumulative Index to Nursing and Allied Health Literature (CINAHL), Scientific Electronic Library Online (SciELO) and Excerpta Medica Database (EMBASE). The findings of each study were summarized and the results were meta-aggregated in JBI SUMARI software.

**Results:**

The search resulted in a total of 447 studies and, after selection, the review included 26 studies, in which 42 nursing strategies were identified. The strategies were first categorised as care- or stewardship-related and then into the subcategories: Screening, Administration, Monitoring and Discharge, Nursing Team, Multi-professional Teams, Patients and Institutional Leadership. The 42 strategies were meta-aggregated and represented in flow diagrams. The best evidence was synthesized related to nursing strategies in the safe management of antimicrobials in the hospital environment.

**Conclusions:**

Nurses play an indispensable function in antimicrobial stewardship in the hospital environment, because they work directly at the core of safe patient care. Significant contributions by nursing towards reducing antimicrobial resistance were found in care-related practice, education activities, research and policy.

## Background

Antimicrobials are drugs that have historically revolutionised treatment for infectious diseases caused by bacteria and reduced world morbidity and mortality rates associated with bacterial infections [1].

In 2018, the World Health Organisation (WHO) collected data on human health-related consumption of antimicrobials in 65 countries and territories. In Brazil, that survey showed mean consumption of 22.75 defined daily doses per 100,000 population, which is higher than in European countries and the highest in the Americas [2].

Excessive use, high cost and improper use of antimicrobials have been growing in the hospital environment and, increasingly, microbial strains resistant to these drugs are developing [3]. In that scenario, antimicrobial resistance has become one of the most important public health problems worldwide [4].

The causation of antimicrobial resistance is considered to be multifactor. However, improper and excessive use of antibiotics in hospital environments are the main contributing factors that have been found to hinder the safe management of antimicrobials [5].

### Statement of the problem

Growing antimicrobial resistance is thus of major concern [6]. One early study, which observed portions of the impact of antimicrobial resistance, showed that if resistance were to continue to increase, this would lead, in 2050, to 10 million deaths per year – in addition to a 2% to 3.5% decline in gross domestic product, which would cost the world around US$ 100 trillion [7].

### Purpose and what was done

At present, the most important strategy for minimising growing drug resistance is management of the rational use of these antimicrobials. Thoughtful, judicial use of these drugs to achieve the best outcomes for individual patients will limit the development of multi-resistant microorganisms and their spread throughout society [8].

Antimicrobial Stewardship Programmes (ASPs) are expected to optimise treatment of infections and reduce antimicrobial-related incidents. Also, these programmes also reduce hospital costs and significantly lower hospital *Clostridium difficile* infection rates and bacterial resistance [9].

Recent publications have found nurses to be essential to antimicrobial stewardship programmes by virtue of their strategic position as mediators of multi-professional team communication, coordination of care and 24 h patient condition monitoring [8].

One of nurses’ main duties in patient care routines is to perform numerous functions critical to successful infection control and prevention and to antimicrobial stewardship programmes. Nurses in Brazilian hospital institutions are in a leadership position as regards furthering health education for patients and society at large as to the importance of antimicrobial stewardship as part of health promotion activities [8].

With that in mind, this study aimed to summarise the best evidence available with regard to nursing strategies for antimicrobial stewardship in the hospital environment.

## Methods

This qualitative systematic review used meta-aggregation in accordance with the methodological recommendations of the Joanna Briggs Institute (JBI) [10]. The protocol was published in the Prospective Register of Systematic Reviews under No. CRD42021224804 and approved as recorded on 6 January 2021.

In keeping with the methodology proposed by the JBI, the Preferred Reporting Items for Systematic Reviews and Meta-Analyses (PRISMA) [11] were used in summarising the evidence about nursing strategies in antimicrobial stewardship in the hospital environment.

As regards potential conflicts of interest, the authors declare that there are no conflicts regarding the planning and execution of this study and that it had no funding of any kind.

Studies and other materials were judged eligible to form part of the systematic review on the criteria that they be studies published from 2016 to 2020 with qualitative observational designs, including interpretative studies, reports and expert technical and scientific opinions, and/or exploratory studies by nurses and/or healthcare personnel of nursing strategies relating to antimicrobial stewardship in the hospital environment. The time frame was set on the rationale that the alert to the crisis in antimicrobial resistance was reported at the World Health Assembly in May 2016, resulting in the Global Action Plan, through to 2020, so as to take in more recent studies. Grey literature was also included. There were no exclusion criteria.

The PICo strategy was used in formulating the review question, where the “P” corresponded to the nursing team; “I”, to the evidence relating to nursing strategies for antimicrobial stewardship and “Co”, to the hospital environment, resulting in the question “What is the best evidence relating to nursing strategies for antimicrobial stewardship in the hospital environment?”.

The relevant literature was identified in the following data bases and journal repositories: Latin American and Caribbean Health Sciences Literature (LILACS) via the Virtual Health Library (VHL), Medical Literature Analysis and Retrieval System on-line (Medline) via PubMed, Cumulative Index to Nursing and Allied Health Literature (CINAHL), Scientific Electronic Library Online (SciELO) and Excerpta Medica Database (EMBASE). The search strategy used in each database is described in Table 1. Searches were conducted on 14 April 2021. For grey literature, the sources were the Open Grey data base and the Proqualis website (proqualis.net). In addition, manual searches were performed from the references cited in the papers selected in the same period.

**Table 1:** Search strategies in the databases selected in the study, 2022

**PUBMED**
(((nursing care[mh] OR nursing care OR nurses[mh] OR nursing[mh] OR nurse* OR nursing*) AND (“drug resistance, microbial”[mh] OR microbial drug resistanc* OR antimicrobial drug resistanc* OR antibiotic resistanc* OR drug resistance, bacterial[mh] OR bacterial drug resistanc* OR antibacterial drug resistanc*)) AND (antimicrobial stewardship[mh] OR antimicrobial stewardship OR antibiotic stewardship)) AND (English[lang] OR Portuguese[lang] OR Spanish[lang])
**EMBASE**
(‘nurse’/exp OR ‘nurse’ OR ‘nurses’ OR ‘nursing’/exp OR ‘nursing’ OR ‘nursing care’/exp OR ‘nursing care’) AND (‘antimicrobial stewardship’/exp OR ‘antibiotic stewardship’ OR ‘antimicrobial stewardship’) AND (‘antibiotic resistance’/exp OR ‘antibacterial drug resistance’ OR ‘antibacterial resistance’ OR ‘antibiotic non-susceptibility’ OR ‘antibiotic nonsusceptibility’ OR ‘antibiotic resistance’ OR ‘antimicrobial drug resistance’ OR ‘antimicrobial resistance’ OR ‘bacterial drug resistance’ OR ‘bacterial resistance’ OR ‘bacterium resistance’ OR ‘drug resistance, bacterial’ OR ‘drug resistance, microbial’ OR ‘microbial drug resistance’ OR ‘resistance, antibiotic’) AND [embase]/lim AND ([english]/lim OR [portuguese]/lim OR [spanish]/lim)
**BVS**
(“nursing care” OR “cuidado de enfermagem” OR “cuidados de enfermagem” OR “atencion de enfermaria” OR nurse* OR nursing OR enfermeir* OR enfermer*) AND (“antimicrobial stewardship” OR “antibiotic stewardship” OR “gestao de antimicrobianos” OR “administração de antibioticos” OR “programas de optimizacion del uso de los antimicrobianos” OR “administracion de antibióticos”) AND ( db:(“LILACS”) AND la:(“pt” OR “es” OR “en”))
**CINAHL**
(((“nursing care” OR nursing OR nurse*) AND (“microbial drug resistance” OR “microbial drug resistances” OR “antimicrobial drug resistance” OR “antimicrobial drug resistances” OR “antibiotic resistance” OR “antibiotic resistances” OR “bacterial drug resistance” OR “bacterial drug resistances” OR “antibacterial drug resistance” OR “antibacterial drug resistances”)) AND (“antimicrobial stewardship” OR “antibiotic stewardship”))
**SciELO**
(“nursing care” OR “cuidado de enfermagem” OR “cuidados de enfermagem” OR “atencion de enfermaria” OR nurse* OR nursing OR enfermeir* OR enfermer*) AND (“antimicrobial stewardship” OR “antibiotic stewardship” OR “gestao de antimicrobianos” OR “administração de antibioticos” OR “programas de optimizacion del uso de los antimicrobianos” OR “administracion de antibióticos”)

Source: The author, 2022

To prevent reporting bias, a comprehensive search was made for studies that met the eligibility criteria for a JBI-based systematic review. The review authors ensured that several data bases were searched. Also, inclusion of data on unpublished studies proved to be a way of reducing publication bias.

After searching the data bases mentioned and other information sources, the findings were exported to the Endnote reference generator, where they were organised for screening.

In this study, screening was carried out using the *Rayyan* [12] software, at first by two reviewers, working independently, by examining abstracts and titles to identify those relevant to the proposed subject. In cases where the data provided were insufficient to decide on inclusion or exclusion of a study, it was read in full, thus avoiding improper exclusions. In the event of disagreement between reviewers, a third was consulted to examine the study and, after discussion, the majority opinion prevailed.

Data were extracted from the papers using an instrument containing the following information: authors, year, title and abstract with the main findings. Qualitative data were extracted from the papers included in the review, using the standardised model data extraction tool JBI-QARI Data Extraction Tool for Qualitative Research [10]. The method chosen to assess the quality of the evidence was the Confidence in the Evidence from Reviews of Qualitative Research (GRADE-CERQual) [13].

All papers selected for inclusion in the systematic review – that is, those that met the inclusion criteria described in the protocol – were subjected to rigorous review by two reviewers working independently. The results from that assessment were used to inform the synthesis and interpretation of the study findings. That review used the JBI Critical Appraisal – Checklist for Qualitative Research for the purpose of assessing each study’s methodological quality and determining to what extent the study addressed the possibility of bias in its design, execution and analysis [10].

One review criterion was that studies should return at least 70% positive responses in the evaluation. When the response was “Not applicable”, the question was not considered in calculating the risk of bias.

Although methodological quality was not assessed to a standard of percentage positive responses, the criterion followed was similar to that used in another study [14], that is, by categorising as high risk those returning up to 49% “Yes” responses, moderate risk those with 50 to 69% “Yes” responses and low risk when returning 70% or more “Yes” responses. Note that this classification was specified in agreement with other authors [14].

The findings of each study were summarised in such a way as to synthesise the evidence identified. An effort was made to condense the research results using the meta-aggregation setting of the JBI SUMARI software.

A record instrument was constructed where the papers were numbered in chronological order with the following information: year of year of publication, author(s), paper title, research objective and findings and/or recommendations.

The studies selected for this systematic review were entered into the SUMARI software and, after inclusion, extracts were taken from each study according to the strategies identified. Each extract was classified by common agreement among the researchers involved in the review, by the levels of credibility set out by the Joanna Briggs Institute. These three levels or degrees of credibility are: Unequivocal (U) – highly credible evidence, not in doubt; Credible (C) – although an interpretation, credible in view of the data; and Unsupported (Un) – findings not supported by the data [10].

After this stage, the passages extracted were categorised by the strategies they expressed. The categories were also summarised into Synthesized Findings, which could be represented in a meta-aggregative flow diagram. These categories were they synthesised to produce a single inclusive set of synthesised findings that could be used as grounds for evidence-based practice.

## Results

The search of studies in the data bases resulted in 447 papers selected. Of these, 72 (16.1%) were excluded as duplicates. After excluding the duplicates, the titles and abstracts of the 375 papers were appraised by the primary and secondary reviewers independently on the inclusion and exclusion criteria of the systematic review.

The reviewers diverged on 105 (28%) of the studies selected. A third reviewer, a specialist in the subject and trained in using the software, was consulted to examine these papers on which there was divergence. The majority opinion prevailed.

After these appraisals, 184 papers were selected, of which 17 (9.8%) were considered losses: 12 were congress summaries or editorials, access to 2 was restricted, 2 were not found in any data base and 1 could not be located because there were several files with the same name (“Antibiotic Stewardship”).

A preliminary selection of 167 papers was organised into a table containing title, year and abstract of each paper. In that way, it was possible to visualise the strategies adopted among the studies and compare the resulting findings.

**Fig. 1:**
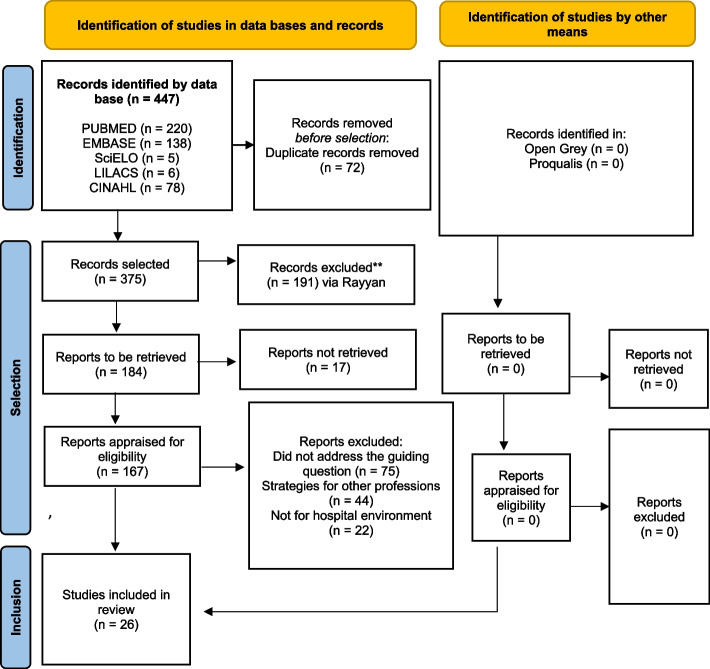
Flow diagram of the search and inclusion process. RJ, 2022. Source: Page (2021) [12]

After reading those papers in full, 75 papers (44.9%) were found not to answer the research question, 44 (26.3%) proposed strategies directed to other professions and 22 (13.2%) were conducted in non-hospital settings. The final sample was thus 26 papers included in keeping with the study eligibility criteria, corresponding to 15.6% of the preliminary selection. The flow diagram is shown in Fig. 1.

The studies were all published in English, except for three in Portuguese [15–17]. The years with the largest numbers of publications were found to be 2018 (*n* = 7) and 2019 (*n* = 7).

By the GRADE CERqual classification, nine studies were found to be reliable at the Unequivocal or Credible levels, meaning that there were “no or very minor concerns as to methodological limitations/ relevance/ coherence/ appropriateness” and “minor concerns as to methodological limitations/ relevance/ coherence/ appropriateness”, equivalent to 69.2% of the studies included in the review (34.6% per level). Six studies (23.1%) were considered to offer low levels of credibility and two, very low levels (7.7%).

The 26 studies included were subjected to methodological quality analysis using the JBI Critical Appraisal Checklist for Qualitative Research instrument, as in Table 2.

**Table 2:** Results of the critical appraisal of studies included using the JBI critical appraisal checklist. RJ, 2022

	References/Year	Q1	Q2	Q3	Q4	Q5	Q6	Q7	Q8	Q9	Q10	Total	Risk of bias
1	Zukowski, C.M., 2016 [18]	Y	Y	Y	Y	Y	Y	N	Y	NA	Y	88.8%	Low
2	Manning, M.L.L., 2016 [19]	Y	Y	Y	Y	Y	Y	N	Y	NA	Y	88.8%	Low
3	Olans, R. et al., 2016 [20]	Y	Y	Y	Y	Y	Y	N	Y	NA	Y	88.8%	Low
4	Cheon, S. et al., 2016 [21]	Y	Y	Y	Y	Y	Y	N	Y	NC	Y	80.0%	Low
5	Jeffs, L. et al., 2017 [22]	Y	Y	Y	Y	Y	Y	N	Y	Y	Y	90.0%	Low
6	Olans, R.D.; 2017 [23]	Y	Y	Y	Y	Y	Y	N	Y	NA	Y	88.8%	Low
7	Sumner, S. 2017 [24]	Y	Y	Y	Y	Y	Y	N	Y	NA	Y	88.8%	Low
8	Alividza, V., 2017 [25]	Y	Y	Y	Y	Y	Y	N	Y	NA	Y	88.8%	Low
9	JAC Antimicrob Resist, 2018 [26]	Y	Y	Y	Y	Y	Y	Y	Y	NA	Y	100.0%	Low
10	Ladenheim, D., 2018 [27]	Y	Y	Y	Y	Y	Y	N	Y	NA	Y	88.8%	Low
11	Carter, E.J. 2018 [28]	Y	Y	Y	Y	Y	Y	N	Y	Y	Y	90.0%	Low
12	Burnett, E., 2018 [29]	Y	Y	Y	Y	Y	Y	N	Y	NA	Y	88.8%	Low
13	Emberger, J. 2018 [30]	Y	Y	Y	Y	Y	Y	N	Y	NA	Y	88.8%	Low
14	Wiley, K.C.; 2018 [31]	Y	Y	Y	Y	Y	Y	Y	Y	NA	Y	100.0%	Low
15	Carrico, R.M. et al., 2018 [32]	Y	Y	Y	Y	Y	Y	N	Y	NA	Y	88.8%	Low
16	Wilson, A., 2019 [33]	Y	Y	Y	Y	Y	Y	N	Y	NA	Y	88.8%	Low
17	Alvim, A.L.S., 2019 [17]	Y	Y	Y	Y	Y	Y	N	Y	NA	Y	88.8%	Low
18	Chater, A.; 2019 [34]	Y	Y	Y	Y	Y	Y	N	Y	NA	Y	88.8%	Low
19	Felix, A.M.S; 2019 [15]	Y	Y	Y	Y	Y	Y	N	Y	NA	Y	88.8%	Low
20	Olans, R.D.; 2019 [34]	Y	Y	Y	Y	Y	Y	N	Y	NA	Y	88.8%	Low
21	Morgan, S.A., 2019 [35]	Y	Y	Y	Y	Y	Y	N	Y	NA	Y	88.8%	Low
22	Hughes, S.J; 2019 [36]	Y	Y	Y	Y	Y	Y	N	Y	NA	Y	88.8%	Low
23	Courtenay M.; McEwen J 2020 [37]	Y	Y	Y	Y	Y	Y	Y	Y	NA	Y	100.0%	Low
24	Courtenay, M. 2020 [38]	Y	Y	Y	Y	Y	Y	N	Y	NA	Y	88.8%	Low
25	Cunha, T.L. 2020 [16]	Y	Y	Y	Y	Y	Y	N	Y	NA	Y	88.8%	Low
26	Kirby E.; 2020 [39]	Y	Y	Y	Y	Y	Y	N	Y	Y	Y	90.0%	Low

Legend: *Q* Question, *Y* Yes, *N* No, *NA* Not applicable, *NC* Not clear

Before the instrument was applied, all the studies were re-read so as to make the appraisal more precise. That reading also reviewed all the categorisations of the care-related and stewardship-related strategies, as well as processing the content of the publications and the findings of the studies.

All the studies showed congruency between their stated philosophical perspective and research methodology (Q1). Only three studies [26, 31, 37] addressed the researcher’s influence (Q7). In all the studies, the participants were represented and the conclusions were based on the data collected, and constructed by way of observation, interviews or other processes (Q8, Q10).

Most of the studies 84.6% (*n* = 22), because they were literature studies, were classified “Not applicable” (NA) as regards whether or not they satisfied ethical concerns or gave evidence of approval by a competent authority. Only one study (3.8%) fitted this criterion and was classed “NC” (Not clear), because the issue was not evident.

**Fig. 2:**
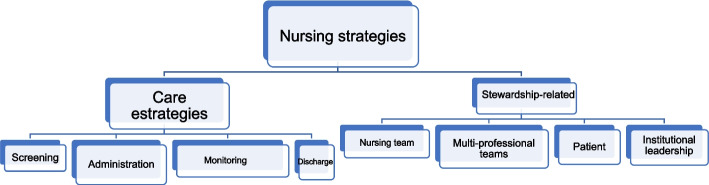
Categorisation of the nursing strategies identified in the studies. RJ, Brazil, 2022. Source: The author, 2022

After these 26 studies had been appraised, they were separated into the care-related and stewardship-related categories and, subsequently, into subcategories by the nursing strategies identified and examined in each study (Fig. 2).

These categories were then subjected to meta-aggregation to produce synthesised findings for the purpose of producing an evidence base for practice and recommendations for further research.

The findings of the qualitative research were grouped using the JBI-QARI Data Extraction Tool for Qualitative Research software, which involved synthesising findings to generate a set of categories that would represent an aggregation. This was possible by joining findings, classified by quality, and categorising them on the basis of similarity of meaning.

These categories were then meta-synthesised to produce a single comprehensive set of summarised findings to underpin evidence-based practice.

**Fig. 3:**
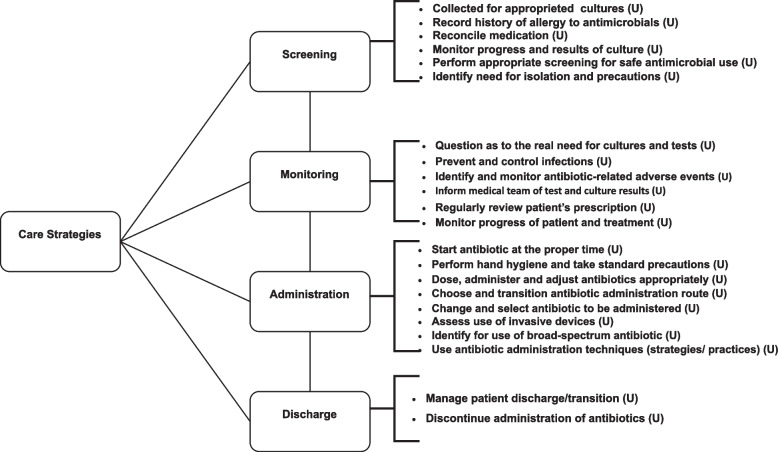
Flow diagram of Care Strategies identified in the meta-aggregation of findings prepared using the Joanna Briggs Institute SUMARI software. RJ, 2022. Legend: U = Unequivocal. Source: the author, 2022

In the care-related category, 22 strategies were meta-aggregated into subcategories including Screening (6 strategies), Administration (8), Monitoring (6) and Discharge (2), by stages in the hospitalisation process (Fig. 3).

**Fig. 4:**
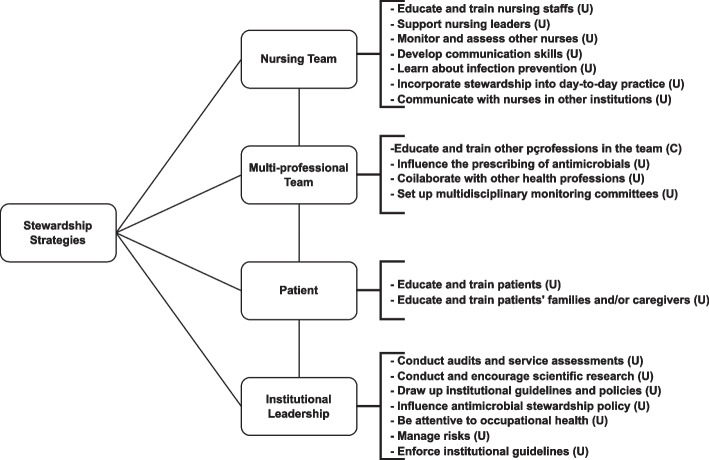
Flow diagram of stewardship strategies identified in the meta-aggregation of findings prepared using the Joanna Briggs Institute SUMARI software. RJ, 2022. Legend: U = Unequivocal; C = Credible. Source: the author, 2022

In addition to the meta-aggregation of care strategies, meta-aggregation was also performed to identify stewardship strategies, Fig. 4.

Note that, by the data surveyed, most of the findings of this systematic review were classified as unequivocal (U), that is, offering strongly credible evidence, while only nine were classified as credible (C). The aggregation thus included only unequivocal and credible findings, which lends reliability to the strategies presented.

## Discussion

As regards the incipient nature of this topic, as reflected in the absence of related grey literature, no stewardship protocols or guidelines were found to steer nursing strategies for the safe use of antimicrobials in the hospital environment.

One study corroborating this finding performed an integrative review to examine how the scientific literature described nurses and their role in Brazil’s antimicrobial stewardship programme (*Programa de Gerenciamento do Uso de Antimicrobianos*, PGUA). During the study period (2013 to 2017), there was a conspicuous lack of Brazilian studies published in the selected data bases, in which international studies predominated [17].

This shows that, although the number of Brazilian studies on the subject is increasing with time, there is still a lack of publications, underlining how much this subject needs to be explored in greater depth.

The studies included in this systematic review were organised in chronological order and the level of confidence in each was evaluated using GRADE-CERQual [13]. Most of the studies (*n* = 18; 69.2%), were considered as meriting high or moderate confidence. Only eight (30.8%) were considered to warrant low or very low confidence.

When a review finding is assessed as deserving of “high confidence”, no further explanation is necessary, because the point of departure for “high confidence” is a situation where each finding of the review can be considered a reasonable representation of the phenomenon in question [40].

The JBI assessment instrument enables researchers to specify criteria for classifying studies into low risk of bias, uncertain risk of bias and high risk of bias; for retaining or excluding assessment criteria; and for retaining or excluding studies of poor methodological quality [10].

Generally speaking, all the studies included in this review recognised nurses to be personnel fundamental to the proper functioning of the antimicrobial stewardship programme – although, in practice, nurses are often not recognised as such, among other things because their activities in such programmes are not described in minute detail.

Although the antimicrobial stewardship programme is interdisciplinary and should contemplate participation by pharmacists, doctors, microbiologists, infection control staff and administrators, nurses have yet to be completely involved. Despite the fact that nurses work directly in the preparation, administration and monitoring of antibiotics, studies have shown that these personnel’s involvement has led to the successful implementation of infection prevention and control practices [10].

Accordingly, in practice, nurses’ routine activities have demonstrated their role in the antimicrobial stewardship programme. Comparison between the activities that constitute antimicrobial stewardship and nurses’ daily routine responsibilities shows overlapping acrtivities [8]. These activities can be seen to be both care related and stewardship related, as in the studies in this systematic review. Also, the guidelines for formulation of the Brazilian health service antimicrobial use management programme (*Programa de Gerenciamento do Uso de Antimicrobianos em Serviços de Saúde*)*,* launched by the national health surveillance agency, ANVISA, in 2017, suggest that the stewardship team include nursing staff representatives [41]. That team should coordinate administrative and general actions, and set up systematic evaluation and oversight to ensure compliance with the plan in the various hospital units. The document also mentions the operational team, which is responsible for the preparation, execution and monitoring of actions of the stewardship programme [17], particularly the care activities.

That division of activities into care-related and stewardship-related was based on Brazil’s nursing code, the *Lei do Exercício da Enfermagem*. In both stewardship-related and care-related areas, nurses’ should supervise the nursing team and its procedures, whether invasive or not [41]. Accordingly, in this study, in order for nurses’ main activities to be addressed clearly, the strategies were categorised into these two approaches – care-related and stewardship-related – as described below.

Nurses’ work comprises various functions, one of the foremost including responsibility for administration of medications. This is one of the care practices most performed in nurses’ day-to-day work, involving administration techniques, communication and patient guidance regarding the medicine being administered, as well as monitoring for possible clinical or treatment-related complications from extravasation or infiltration of drugs administered [42].

Administration is a procedure demanding professional knowledge and competence, in which nurses must see patients receiving medication not only from the biological standpoint, but as fellow beings interacting with the nurse at the time they receive the medication, which embodies the opportunity to regain their health [43].

It is important to stress that this involves not only technical skill; other aspects must be borne in mind in order for administration to be successful. Drug administration is an important nursing practice, so that nurses must be familiar with all the aspects involved. It is an activity requiring, on the nurse’s part, current technical knowhow and a critical outlook, given that this forms part of nurses’ and nursing teams’ essential clinical skills and poses risks to patients and nurses thjemselves [44].

Nurses’ perform activities at the various stages of drug administration, always showing concern for patient safety by controlling timetables, dilutions and appropriate intervals. It is their duty to take action with a view to improving safety, minimising errors and ensuring effective treatment [45].

Another subcategory with a significant number of mentions was Patient Screening, with 60 mentions in the studies examined.

On arrival at hospital, patients are screened and placed under appropriate precautionary care. This function is performed by nurses in the institution’s screening sector or by nurses of the hospital admissions staff. The patient’s history of allergy to medications is assessed [23].

Appropriate screening directed to safe antimicrobial management forms part of the nurses’ work during hospital admission. Patients should be asked about any history of allergy to drugs, especially antibiotics and about signs and symptoms of such allergies [28].

Another subcategory, Patient Monitoring for antimicrobial resistance, received 44 mentions. Nurses’ presence and responsibility in patient monitoring underscore their importance in detecting and recording changes in patient condition [40].

Nurses’ key contribution to the antimicrobial stewardship programme is to guarantee the safety and quality of healthcare monitoring associated with infections and adverse antimicrobial-related events, mainly in countries such as Canada and the United States. Specifically, nurses play a fundamental role in identifying adverse events such as cutaneous eruptions and diarrhoea associated with antimicrobials, monitoring and fostering adherence to institutional guidelines and best practices in antimicrobial use, educating patients about the antimicrobial stewardship programme and minimising the need for microbiology testing [22].

The “Discharge” subcategory returned only 17 mentions, fewer than the other care subcategories.

Nurses are the care professionals who manage and plan discharge, because they are in the hospital continuously, serving as the link between patient and the other professions of the multi-professional team, in addition to being those most engaged in research into discharge planning [46].

During discharge, it is important that patients/caregivers receive guidance as regards any antibiotics administered during hospitalisation and also in relation to those to be used at home following discharge. Discharge instruction documents will have to be updated to reflect the concepts of antimicrobial stewardship, and nurses will have to be trained to teach patients those concepts effectively and knowledgeably during discharge. For example, teaching for patients being discharged with an antibiotic should be standardised. Current standardised teaching materials must be reviewed, because antibiotics have now been included [24].

In the stewardship-related category, it was the “Nursing team” subcategory that returned most mentions, a total of 55.

One focus in relation to the nursing team was educational activities. These are designed to build team capacity by upgrading techniques and knowledge of new technologies. Accordingly, as a methodological proposal, continued professional development for nurses is considered to be an important care-related practice. The content addressed should thus be directed to the realities of each institution and consider day-to-day working conditions, sector needs and quality indicator findings as regards safe management of antimicrobials [47].

However, health education does not depend solely on nurses individually; other contributing factors include the institution and team involved. Education for nurses should aim to bring about change in day-to-day care, and enter the work process institutionally through a commitment between workers and users, connection among management, adherence to new technologies, equipment, staffing, materials, medication and education measures that permits overall personnel development work process, constructed collectively and established as a key strategy for achieving institutional and individual development outcomes [47].

The “Multi-professional teams” subcategory returned the second-largest number of mentions (*n* = 46).

Note that antimicrobial stewardship is a multidisciplinary endeavour that concentrates on reducing high rates of improper antimicrobial use [26]. ASP guidelines repeatedly stress that broad multidisciplinary involvement is essential to attaining the objectives of antimicrobial stewardship [23].

This subcategory also highlights the importance of setting up infection prevention and control committees and protocols, using antibiogram data, measuring and monitoring antibiotic use, applying epidemiological surveillance indicators of healthcare-related infections, conducting surveillance at the conclusion of antimicrobial administration and offering capacity-building for health personnel.

Infection prevention requires comprehensive involvement by patients, relatives and caregivers. In that regard, patients must be enabled to perform self-care so as to minimise the risk of avoidable incidents and harm. They often trust to their relatives and others for care. Patient involvement starts with shared information, assessment of the patient’s ability to perform the required tasks so as to ensure they are able to perform those tasks, having an assessment of that performance and giving feedback about possible improvements [32].

When talking about antimicrobial stewardship it is important to involve patients in conversations about the best approach to their treatment, focusing on how this can be achieved, suggesting manners of attaining good health and planning the path to be taken [48].

Overall, education of patients and caregivers is a key aspect of the antimicrobial stewardship programme [24].

One strategy would be to form nursing organisations and groups for the purpose of suggesting and promoting nurses’ participation in national committees and agencies involved with antibiotic management and related policies [21].

In Brazil, Health Surveillance Agency (Anvisa) Resolution (*Resolução da Diretoria Colegiada*, RDC) No. 7 of 2010 and the National Healthcare-related Infection Prevention and Control Programme (*Programa Nacional de Prevenção e Controle de IRAS*, PNCIRAS) for the four-year period 2016–2020, both recommend that health institutions take steps to reduce and control the incidence of multi-resistant microorganisms [49].

## Limitation

This review also highlighted the fact that there are few – and particularly few Brazilian – publications on nurses’ importance to the programme, which posed a limitation on this study. Also, although many studies addressed nursing strategies, nurses’ role is still not recognised in the world’s main guidelines on antimicrobial stewardship, which prevented more robust evidence from being presented in this study.

## Conclusion

This systematic review identified, categorised and meta-aggregated 42 nursing strategies for improving safety in the use of antimicrobials. Prominent among the care strategies were the subcategories Screening, Antimicrobial Administration, Monitoring and Discharge and in the stewardship subcategories, Nursing Teams, Multi-Professional Teams, Patients and Institutional Leadership.

It was found that nurses’ routine activities already form part of the antimicrobial stewardship programme. The nursing profession is directly concerned with patient safety and makes significant contributions in practice, education, research and policy focused on reducing antimicrobial resistance.

Accordingly, although there are numerous strategies to be explored and major opportunities in the field of nursing, it is recommended that the issue be studied in greater depth to identify that recognition, given the fundamental role played by nurses in antimicrobial stewardship programmes.

## Data Availability

All the material is owned by the authors and are available from the corresponding author upon reasonable request.
